# The state of surgery, obstetrics, trauma, and anaesthesia care in Ghana: a narrative review

**DOI:** 10.1080/16549716.2022.2104301

**Published:** 2022-08-12

**Authors:** Desmond T. Jumbam, Emmanuella Amoako, Paa-Kwesi Blankson, Meredith Xepoleas, Shady Said, Elikem Nyavor, Adam Gyedu, Opoku W. Ampomah, Ulrick Sidney Kanmounye

**Affiliations:** a Department of Policy and Advocacy, Operation Smile Ghana, Accra, Ghana; b Department of Policy and Advocacy, Operation Smile, Virginia Beach, Virginia, USA; c Department of Paediatrics and Child Health, Cape Coast Teaching Hospital, Cape Coast, Ghana; d Department of Paediatrics and Child Health, Korle-Bu Teaching Hospital, Accra, Ghana; e Oral and Maxillofacial Surgery Unit, Korle-Bu Teaching Hospital, Accra, Ghana; f Department of Surgery, School of Medicine and Dentistry, Kwame Nkrumah University of Science and Technology, Kumasi, Ghana; g Department of Surgery, University Hospital, Kwame Nkrumah University of Science and Technology, Kumasi, Ghana; h Plastics and Reconstructive Surgery Unit, Korle-Bu Teaching Hospital, Accra, Ghana

**Keywords:** Ghana, global surgery, health system strengthening, National Surgical Obstetrics and Anaesthesia Plan, policy analysis

## Abstract

**Background:**

Conditions amenable to surgical, obstetric, trauma, and anaesthesia (SOTA) care are a major contributor to death and disability in Ghana. SOTA care is an essential component of a well-functioning health system, and better understanding of the state of SOTA care in Ghana is necessary to design policies to address gaps in SOTA care delivery.

**Objective:**

The aim of this study is to assess the current situation of SOTA care in Ghana.

**Methods:**

A situation analysis was conducted as a narrative review of published scientific literature. Information was extracted from studies according to five health system domains related to SOTA care: service delivery, workforce, infrastructure, finance, and information management.

**Results:**

Ghanaians face numerous barriers to accessing quality SOTA care, primarily due to health system inadequacies. Over 77% of surgical operations performed in Ghana are essential procedures, most of which are performed at district-level hospitals that do not have consistent access to imaging and operative room fundamentals. Tertiary facilities have consistent access to these modalities but lack consistent access to oxygen and/or oxygen concentrators on-site as well as surgical supplies and anaesthetic medicines. Ghanaian patients cover up to 91% of direct SOTA costs out-of-pocket, while health insurance only covers up to 14% of the costs. The Ghanaian surgical system also faces severe workforce inadequacies especially in district-level facilities. Most specialty surgeons are concentrated in urban areas. Ghana’s health system lacks a solid information management foundation as it does not have centralized SOTA databases, leading to incomplete, poorly coded, and illegible patient information.

**Conclusion:**

This review establishes that surgical services provided in Ghana are focused primarily on district-level facilities that lack adequate infrastructure and face workforce shortages, among other challenges. A comprehensive scale-up of Ghana’s surgical infrastructure, workforce, national insurance plan, and information systems is warranted to improve Ghana’s surgical system.

## Background

Surgical conditions remain a significant, yet largely underestimated source of the global burden of disease. Approximately five billion people worldwide lack access to safe, timely, and affordable emergency and essential surgical care [1]. The largest capacity deficit is in low- and middle-income countries (LMICs), where the poorest one-third of the world’s population receives only 3.5% of all surgical procedures conducted globally [2]. The inadequate supply of quality surgical care takes a serious human and economic toll, exacerbating acute, life-threatening complications and leading to increased disability and death [3]. Approximately 28% of the global disability-adjusted life years (DALYs) lost yearly results from surgical conditions [4]. In Sub-Saharan Africa (SSA), surgical conditions account for 25 million DALYs lost each year [5].

The care that is needed to manage surgical conditions – surgical, obstetric, trauma, and anaesthesia (SOTA) – is relatively complex, requiring a synergy between all pillars of the healthcare system including infrastructural foundations, such as running water, electricity, skilled staff, and more. Many health systems in SSA have inadequate infrastructure, human resources, and systems needed for SOTA care. Furthermore, national health plans fail to prioritize SOTA care, leading to surgically treatable conditions such as obstructed labour [6,7], traumatic injuries [7,8], and congenital conditions [9,10] to remain frequently untreated in LMICs [11].

Ghana is a lower-middle-income country located in SSA with a population of 30 million people [12]. While much progress has been made in Ghana since its independence to improve health care delivery and public health, access to quality emergency and essential SOTA care has remained low for many Ghanaians. Currently, Ghana’s healthcare system is unable to meet the healthcare needs of the population; 4.6% of children die before reaching the age of 5 years, and the maternal mortality rate of 308 per 100,000 live births is higher than global targets [13]. Additionally, the Ghanaian health system meets only about 12% of the estimated trauma surgical need in the country [14]. The Lancet Commission on Global Surgery (LCoGS) recommends 5,000 surgical procedures per 100,000 population as a target to meet population surgical needs [1]. The current surgical volume rate in Ghana is 869 surgical procedures per 100,000 population, reflecting the health system’s inadequacies and inability to meet the surgical needs of the population [15]. Ghana’s surgical system needs to be strengthened to meet the surgical needs of Ghanaians and reduce the high mortality and morbidity from surgical conditions.

## Rationale and aims of this review

**Figure 1.**
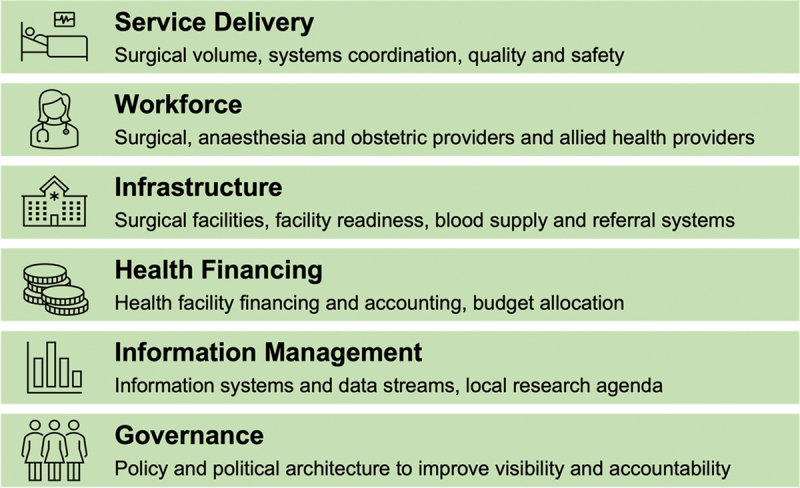
The six domains of the national surgical system framework adapted from the lancet commission on global surgery and WHO health systems building blocks [16].

The nature of SOTA care delivery requires a concerted and coordinated strategy to scale up SOTA care to meet the needs of the Ghanaian population. A comprehensive understanding of the surgical landscape is needed to design holistic strategies and policies to scale up SOTA care nationally. While individual studies assessing separate aspects of the surgical system are beneficial, a comprehensive summary of these studies is needed to gather a holistic picture of the strengths and gaps of the entire surgical system. Thus, we conducted a situation analysis to identify the strengths and weaknesses of the SOTA landscape in Ghana using a narrative literature review of published studies. This narrative review aims to provide an overview of the surgical landscape through the World Health Organization’s health system framework of five of the six domains: service delivery, workforce, infrastructure, finance, and information management (Figure 1).

## Methods

We conducted a narrative review by searching the PubMed database using the following keywords: surgery, anaesthesia, obstetrics, trauma, and Ghana. Variations of these keywords were searched in combination with each other. PubMed is a database that includes literature from Medline, life science journals as well as online books. The search was limited to research articles published in the English Language between 2005 and 2020. Articles included in the review specifically focused on SOTA capacity, delivery, challenges, and strengths in Ghana. Studies that did not focus on the Ghana surgical system were excluded.

**Figure 2.**
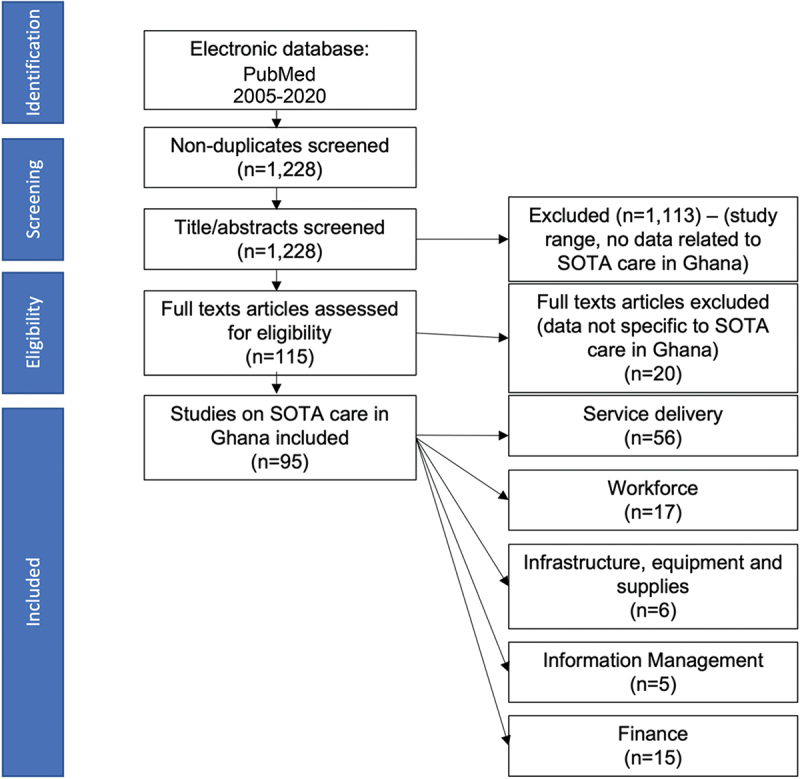
Flowchart of the search and screening process.

Articles identified through the search were downloaded, and titles were screened by two authors using an online systematic review platform Rayyan (Rayyan Systems Inc., Cambridge, MA, USA) [17]. The authors then reviewed the abstracts and full articles of all studies that met the inclusion criteria. In total, 95 studies were deemed to contain information useful to the objectives of the review after full text assessment (Figure 2). All studies were categorized into one of the five domains of the health system depending on the area of focus of each article. Fifty-six studies included information on service delivery; 17 on workforce, 6 on infrastructure, equipment, and supplies; 5 on information management, and 15 on health financing. Relevant data including author information, type of study, location of study, and key findings of the study were recorded in a Microsoft Excel document in one of the five domains: service delivery, workforce, infrastructure, information management, and finance (Supplementary file 1). A summary of the key findings of the narrative review is provided in Table 1.

**Table 1.** Summary of the key findings.

Domain	Strengths	Gaps
Service Delivery	• District hospitals provide a majority proportion of emergency and essential SOTA care	• Surgical volume (869 procedures per 100,000 pop) is significantly below recommended target (5,000 per 100,000 pop)
	• Obstetrics and gynecology surgery account for the majority of SOTA provided in Ghana	• Access to pediatric surgical care is severely limited with only about 30,000 pediatric surgical conditions conducted annually
	• Anaesthesia capacity is improving at tertiary hospitals	• Ghana has a cesarean section rate of 7.2%, below WHO recommended rates
	• Anaesthesia-related mortality found to be low at Komfo Anoyke Teaching Hospital	• Trauma is caused by crashes (39%) and falls (20%)
		• 80% of mortality from trauma occur in the field
		• Access to closed fracture reduction and orthopedic procedures are limited at district hospitals
	• Health provider job satisfaction was found to be high at Volta River Authority Hospital	• Local anaesthesia was found to be underused in hospitals in the Northern region
		• 12% of surgical patients at tertiary hospitals develop a surgical site infection
Workforce	• Strong surgical training programs through the Ghana College of Physicians and Surgeons (GCPS) and West African College of Surgeons	• Limited data on surgical workforce in Ghana
		• Most graduates of the GCPS work in Accra and Kumasi, the two largest cities – maldistribution of surgical workforce around the country
	• Most graduates are employed by the Ministry of Health, Ghana Health Service or Christian Health Association of Ghana	• Surgeons have a significant workload
		• Most district hospitals do not have fully trained surgeons
	• Trained surgeons tend to stay at their primary assignment site	• There is a significant shortage of skilled surgical providers, particularly anaesthesia providers
		• Limited training opportunities for sub-specialized surgery available in country
Infrastructure equipment and supplies	• Majority of health facilities have access to reliable water (79%) and electricity (82%)	• Most district hospitals do not have access to consistent imaging and operation room infrastructure
	• Tertiary hospitals have consistent access to imaging and operating room infrastructure	• Unreliable supply of oxygen has been noted at some tertiary facilities
	• Centers for equipment repair exists in most hospitals	• Delayed reimbursement for supplies leads to stock-outs of supplies
		• Infrastructure and supplies deficit is most acute for pediatric surgeries at all levels of care
		• Centers for equipment repair are often understaffed
Information Management	• Electronic information systems are used in tertiary hospitals	• Scarcity of studies on information management around SOTA care
	• District Health Information Management Systems at district and regional hospitals expected to improve data quality	• There is no centralized SOTA database
		• Information sources from logbooks and patient files often incomplete or poorly coded
		• Information of quality of surgery including post-operative mortality rates are not nationally recorded
Finance	• 68% of the Ghanaian population are insured by the National Health Insurance Scheme (NHIS)	• Most of the direct cost to patients is paid out-of-pocket (up to 91%), by charitable organizations (up to 60%) and health insurance (up to 14%)
	• NHIS reduces risk of catastrophic expenditure for surgical care by up to 70%	• Uninsured patients experience catastrophic expenditure than insured patients (29% vs 7%)
	• Risk of catastrophic expenditure is slowly declining	•Women are more than twice likely to experience catastrophic health expenditure than men

## Service delivery

Fifty-six studies focused on SOTA service delivery. In general, these studies revealed significant deficits in SOTA service delivery in Ghana. The amount of surgical care provided is not enough to meet the high need in the country, particularly in rural areas. Although they are generally understaffed and underequipped, district hospitals provide a majority of essential and emergency surgical procedures in Ghana. The national surgical volume rate of Ghana is 869 surgical procedures per 100,000 population compared to the recommended LCoGS target of 5,000 surgical procedures per 100,000 population [15]. Of the surgical procedures performed, over three quarters (77%) are classified as essential surgical procedures according to the classification of the Disease Control Priorities III (DCP3) [5]. The DCP3 has designated 44 surgical procedures as ‘essential’ as they are considered to be the most cost-effective with the highest population impact [5]. This is the definition of essential surgery adopted by most of the studies in this review. Essential procedures account for 64–83% of hospitals’ annual surgical output at any level, and 55% of all essential interventions are emergency procedures [18,19]. Obstetrics and gynaecology procedures make up 47% of essential emergency procedures; 68% of these are caesarean sections [19]. Also, 22% of essential emergency surgeries are general surgical procedures, most of which are wound/abscess care procedures and laparotomies [19].

District hospitals play a critical role in the delivery of SOTA care, particularly emergency and essential SOTA care. One survey found that district hospitals perform 62% of all surgical interventions in Ghana and 83% of these operations are essential surgeries [18]. Of note, faith-based hospitals provide 35% of all operations performed at the district level. This corresponds to 34% of district-level hospitals’ essential surgical procedures and 22% of annual surgical volume. Faith-based hospitals provide 42% of operations outside of the essential category, which are performed by district-level hospitals.

On the other hand, regional hospitals perform only 9% of the national surgical volume. Regional hospitals consistently perform fewer operations than district-level and tertiary hospitals for all operations except colostomy, the establishment of a surgical airway, and the release of urinary obstructions. Thirty-six percent of tertiary hospitals’ annual surgical output is specialized surgical interventions outside of the essential category [18].

### Paediatric surgery

Access to pediatric SOTA care has been shown to be severely lacking in many parts of Africa [8,20–22]. Similarly, studies examining paediatric SOTA care in Ghana indicate that access to paediatric SOTA care is more limited than access to care for surgical conditions affecting adults. About 30,000 paediatric surgical interventions are performed each year yielding a paediatric surgical volume rate of 284 paediatric surgical procedures per 100,000 Ghanaian children [23]. Two-thirds of these interventions are essential procedures, with most paediatric procedures outside of the essential category performed at tertiary facilities (55%) [20]. This is likely because most of the few paediatric surgeons available in Ghana are located at tertiary hospitals in urban areas [24]. The most prevalent paediatric surgeries are abdominal (29%) and are indicated for typhoid perforation in children aged ten years or less [23,25]. While seven out of ten Ghanaian children (72%) with typhoid perforation are male, males appear to have a lower mortality rate than females (7.3% vs. 27%) [26]. Twenty-one percent of paediatric surgery interventions are trauma-related [23]. Household injuries are the most common causes of paediatric trauma. Up to 30% of children sustain a household injury, i.e. an annual incidence rate of 594 per 1,000 children [27]. These are generally due to falls (54%) or cuts (20%) [27]. Fortunately, most injuries are moderate (94%) [27]. Nearly three percent (2.8%) of paediatric surgeries are performed to correct congenital malformations [18]. The mortality rate among neonatal cleft lip and palate surgery can be as high as 25% [28]. This high mortality rate is primarily due to inadequate preoperative preparation, given as most cleft and lip palate neonates suffer from malnutrition [28]. The unique needs of paediatric surgical patients necessitate careful attention during policy and strategy design. Policy makers should ensure that these needs are carefully considered during policy development.

### Geriatric surgery

Surgical care for elderly patients is also not widely performed in Ghana. Geriatric patients (aged ≥65 years) account for a small proportion (7%) of Ghana’s annual surgical volume yielding a national average of 768 geriatric surgical procedures per 100,000 elderly Ghanaians. This corresponds to 15% of the surgical need in this population. About three quarters (74%) of operations in the elderly population are essential surgeries, and the most prevalent surgeries are catheterizations for urinary obstructions (80%). Furthermore, fifty-eight percent of geriatric surgeries are performed at district-level facilities [29].

### Obstetrics and gynecology surgery

Obstetrics and gynaecology surgery account for the majority of SOTA care provided in Ghana. This is similar for other African countries like Tanzania and Nigeria [30,31]. About 90,044 obstetrics and gynaecology surgeries are performed annually in Ghana (39% of the annual surgical volume), giving a surgical volume rate of 881 surgical procedures per 100,000 women per year. Most interventions are essential (87%) and performed at district-level hospitals (71%) [13]. The national caesarean delivery rate is 7.2%, suggesting limited access to emergency and essential obstetric care when compared to World Health Organization-recommended caesarean section rates [32]. These findings indicate that although obstetrics and gynaecology procedures account for most surgical procedures in Ghana, the need for quality obstetrics and gynaecological procedures remains high in the country, particularly in rural areas. District hospitals, where most of these procedures are performed, need to be strengthened to capacity to provide quality obstetrics and gynaecology procedures. This will likely reduce mortality and morbidity resulting for maternal conditions.

### Trauma and orthopaedic surgery

About 35,797 trauma surgeries are performed annually (15% of the annual surgical volume), i.e. an annual trauma operation rate of 134 per 100,000 Ghanaians [14]. District-level facilities perform 38% of the national trauma surgery volume, which hints towards an unmet trauma surgery need in rural Ghana [14]. Trauma in Ghana is caused by road traffic accidents (39%) and falls (20%), and trauma patients are generally young (mean age 27, SD 17 years) and male (68%) [33]. One-third of Ghanaian trauma patients have injury to the head region, and the mortality rate of trauma patients in tertiary facilities is less than 1% [33]. However, most trauma deaths occur outside of healthcare facilities, with 80% of deaths recorded in the field [].

Ghanaian facilities at all levels do not have dependable access to prosthetic and neurosurgical care [34,35]. Of note, access to closed fracture reduction and orthopaedic procedures is inconsistent in district-level facilities; outside tertiary facilities, external fixation for fractures, vascular repair, and skin grafting are rarely offered [35].

Ghana’s ambulance services appear to be more established than ambulance services in other LMICs []. The National Ambulance Service was founded in 2004 and expanded rapidly in its first decade to cover 81% of Ghana [36]. Most of the National Ambulance Service’s activities are inter-hospital transfers (up to 80%), while the rest are roadside interventions [36]. Yet Zakariah et al. note that further improvements are needed including increasing public awareness of ambulance services, strengthening ties to local health facilities, and ensuring appropriate and timely transfers [36].

### Anaesthesia

There was a dearth in studies focused on anaesthesia services in Ghana especially at the regional and district hospital levels. Most studies identified were limited to tertiary institutions. At Komfo Anokye Teaching Hospital (KATH) in Kumasi, one study found that about 10,319 anaesthetic procedures are performed annually and 45% of these anaesthetic procedures are for emergency surgeries [37]. Post-anaesthetic care units (PACUs) were linked to all 16 operating rooms, and the department of anaesthesia oversaw a six-bed critical care unit. Most anaesthesia procedures were general anaesthesia (54%) and spinal block (42%). Local anaesthesia (3%) and peripheral nerve block (1%) are less common. The most prevalent surgical interventions are obstetrics and gynaecology (29%), general surgery (17%), and orthopaedic surgery (16%). More than eight in ten obstetrics and gynaecology surgeries are caesarean sections (82%). Of note, anaesthesia providers do not deliver labour analgesia [37]. One study assessing the use of local anaesthesia in inguinal hernia repairs in Northern Ghana concluded after assessing 8,080 patients that local anaesthesia was underused in Northern Ghana [38]. The paucity of studies assessing anaesthesia services in Ghana, especially at district and regional hospitals, may be an indication of the low priority afforded to anaesthesia care nationally. There is a need to study the anaesthesia need and quality of services provided at lower-level facilities to design policies to strengthen anaesthesia services nationwide. Recently, the College of Anaesthesiologists of East, Central and Southern Africa (CANECSA) was created to meet the anaesthesia needs of East, Central, and Southern Africa [39]. The faculties of anaesthesia within the WACS and GCPS could be strengthened to address anaesthesia needs in Ghana and West Africa.

### Quality and safety

Studies on perioperative mortality rates (POMRs) in Ghana are limited and when available, are focused solely on tertiary facilities. Brouillette et al. found the all-cause post-operative mortality rate at KATH in Kumasi to be 0.65%, equating to 1 death per 154 anesthetics [37]. This mortality rate was found to be similar to that of other LMICs like Liberia (1 in 74) [40] and Malawi (1 in 504) [41, 42]. This is, however, significantly higher than that in high-income countries (HICs) where anaesthesia-related mortality can be as low as 1 death in 47,800 hospital discharges in the USA [35]. However, they found mortality in the post-anaesthesia care unit (PACU) to be significantly higher than mortality in the operating room [37]. This higher PACU mortality rate was attributed to critically ill patients being managed in under-resourced PACU for long periods of time.

More than one in ten patients (12%) who undergo surgery at tertiary hospitals develops a surgical site infection (SSI) [43]. Most SSIs are diagnosed during hospitalization (51%), but 49% are diagnosed after discharge [44]. Mortality among patients with surgical site infections is two times higher than among patients without SSIs (8% vs. 3%) [43,44]. Most of the studies conducted on quality of SOTA care in Ghana were conducted at tertiary hospitals. Hence, the findings presented here may not be generalizable to lower-level hospitals. Further research on quality of care at regional hospitals and district hospitals is needed to better understand the quality of SOTA care provided nation-wide.

### Referral

An effective national referral system is crucial for ensuring that patients who need specialized care are escalated up the referral pathway to receive quality care [45]. It is also necessary for ensuring that available resources are used efficiently. We found two studies that documented surgical referrals in Ghana. One study assessing referrals at ten district hospitals in ten different regions found that up to 40% of Ghanaian health facilities refer patients in need of major surgical interventions. The principal reasons for referral are lack of surgical workforce, limited skills, or infrastructure at the referring facility [13]. Another study assessing the quality of referrals at KATH found that referrals for elective surgery came primarily from other teaching hospitals (45%) and district-level facilities (26%) [46]. Most referrers were physicians (66%), while physician/medical assistants and nurses/midwives referred 14% and 2%, respectively. Many district-level facilities lack personnel to offer emergency SOTA care at night and on weekends [46]. In Tanzania, one study found that up to 35% of surgical referrals were preventable and reasons for these preventable referrals were similar to those found in this review [45]. These findings indicate that there are avoidable referrals for procedures that could be performed at lower-level facilities to tertiary specialized facilities. Improving surgical capacity at district and regional hospitals will probably result in fewer avoidable referrals to tertiary hospitals and more efficient use of limited resources.

Aside reducing avoidable referrals, efficient coordination and communication between hospitals is needed to ensure quality care for referred patients. We found evidence of poor coordination in surgical referrals in Ghana. The Ghana Health Service referral form’s utilization decreases essential information’s missingness (17% vs. 50%) [47]. Unfortunately, the utilization of structured referral forms does not eliminate missingness, hinting towards user education. Of note, physician/medical assistants record a working diagnosis more often than nurses/midwives and physicians (96% vs. 93% vs. 75%) [47]. This highlights a need for further education of healthcare providers on the proper use of GHS-structured referral forms to ensure the effectiveness of the surgical referral system.

### Barriers to care

Surgical patients in Ghana face numerous barriers to getting quality SOTA care. These barriers include barriers at the community, systems, and capacity level and address three barriers to care: barriers to seeking, reaching, and receiving quality SOTA care. Insufficient patient education and perception of safety and surgery quality have been found to be significant barriers to seeking SOTA care in Ghana [48]. One study found that the average delay from onset to presentation for non-emergency SOTA conditions is 22.1 months. Patients often seek care at under-resourced facilities (56%) and from traditional or religious healers (43%) before reaching the definitive care facility [49]. These lead to further delays in receiving timely SOTA care. The median time to a facility capable of providing essential surgical services was 62 minutes, well below the 2-hour mark established by LCoGS [50]. However, when patients arrive at healthcare facilities, they often encounter delays in receiving care. For example, the mean admission-to-evaluation time for obstetric patients was found to be as high as 40 minutes [51]. Women find it more difficult to navigate the healthcare system; however, they face fewer barriers to care than men [48]. Patient delays in receiving care at healthcare facilities are often due to system capacity challenges such as limited health workforce, deficient infrastructure equipment and supplies, and cost of care. These barriers to care encountered by Ghanaian patients are further elaborated in the following sections.

## Workforce

We found 17 unique studies related to the surgical workforce in Ghana. While there are several studies that assessed various aspects of the surgical workforce in Ghana, we found that no study provided a comprehensive surgical workforce density for Ghana. It was therefore not possible to compare this with the LCoGS recommended target of 20 specialist surgical providers per 100,000 population. Nonetheless, the studies pointed to a paucity of skilled surgical and anaesthesia care providers of all cadres. For example, in 2016, Ghana had only 70 anaesthesia physicians and 565 nurse anaesthetists for a population of 28.5 million people at the time [37]. Most of the anaesthesia physicians are located in the two biggest cities, Accra and Kumasi. Ghanaian surgeons can either train in Ghana College of Physicians and Surgeons (GCPS) programs or West African College of Surgeons (WACS) Programs [15]. GCPS trainees tend to be more interested in general surgery, trauma and orthopaedics, and urology, and most graduates are employed by the Ministry of Health, the Ghana Health Service, or the Christian Health Association of Ghana (90%) [15]. Fifty-six percent of GCPS graduates work in Accra or Kumasi, the two most populous cities, and one in five graduates work in underserved communities.

The concentration of Ghanaian surgeons in urban areas is higher for niche specialties. For example, there are 11 paediatric surgeons in Ghana and none practice in the Central region [24]. It is worth mentioning that Ghanaian surgeons tend to stay at their primary assignment site. For instance, GCPS programs boast an 87% retention rate [15]. This contrasts starkly to the 1990s when up to 68% of Ghanaian-trained physicians and nurses left the country [,^52^]. Surgeons have a significant workload. On average, they consult approximately 48 patients and perform about 19 surgeries (7 elective and 12 emergency surgeries) each week [15].

### Task-shifting/sharing

Like other countries in SSA, the use of task-sharing and task-shifting for surgery and anaesthesia is very common [53,54]. In fact, our review indicates that the majority of SOTA care in Ghana is provided by task-sharers. District-level facilities contribute to 62% of the national surgical volume, but most do not have fully trained surgeons [18]. These facilities are often staffed by medical officers whose time is spent in delivering SOTA care (i.e. 25% on obstetrics and gynaecology, 13% on non-trauma surgical, and 5% on trauma care) [55].

In 2006, sixty-five percent of anaesthesia care was delivered by nurse anaesthetists, 32.8% by surgeons, and 2% by physician anaesthetists [56]. Ghanaian nurse anaesthetists can train at one of three two-year Bachelor of Science programs: Komfo Anokye Teaching Hospital in Kumasi, Ridge Regional Hospital in Accra, and Tamale Teaching Hospital in Tamale. Only nursing school graduates with two or more years of experience are eligible for admission into the nurse anaesthetists programs [37]. Task-sharing has been impactful to providing surgical care in Ghana. However, this review did not find studies assessing the regulation and quality of care provided by task-sharers. Further studies are needed to assess surgical and anaesthesia services provided by surgical task-sharers. It is also important that policy makers introduce the right regulatory policies to ensure standardization of training offered to task-sharers as well as scope and quality of care provided.

### Training

In Ghana, surgeons, obstetricians, and anaesthesiologists can train in institutions accredited by the GCPS or with the West African College of Surgeons (WACS). The GCPS has eight non-surgical and seven surgical programs [57]. On the other hand, the West African College of Surgeons has four programs in Ghana with a maximal SOTA training capacity of 467 (Table 2) [58].

**Table 2.** Accredited centres of the West African college of surgeons in Ghana offering surgical, obstetric, trauma, and anaesthesia post-graduate training [58].

Institution	Specialty	Resident capacity	Accreditation
Korle Bu Teaching Hospital	Anaesthesia Cardiac surgery Ear, nose, and throat surgery General surgery Neurosurgery Obstetrics and gynecology Ophthalmology Orthopedic and trauma surgery Pediatric surgery Plastic and reconstructive surgery Urology Oral and maxillofacial surgery	60 10 13 15 8 40 39 10 8 8 10 6	Full Full Full Full Full Full Full Full Full Full Full
Komfo Anokye Teaching Hospital	Anaesthesia Ear, nose, and throat surgery General surgery Neurosurgery Obstetrics and gynecology Ophthalmology Orthopedic and trauma surgery Pediatric surgery Plastic and reconstructive surgery Urology Oral and maxillofacial surgery	20 15 35 3 50 11 8 10 3 8 8	Full Full Full Full Full Full Full Full Full Full
Military Hospital Accra	Anaesthesia Ear, nose, and throat surgery General surgery Neurosurgery Obstetrics and gynecology Ophthalmology Orthopedic and trauma surgery Plastic and reconstructive surgery Urology	12 2 3 2 20 6 3 2 3	Full Partial Full Partial Full Partial Partial Partial Partial
FOCOS Orthopaedic Hospital	Anaesthesia Orthopaedic and trauma surgery	12 4	Full Partial

### Job satisfaction

Our review indicates that little research is conducted around job satisfaction in Ghana. One study found job satisfaction to be high among Ghanaian healthcare providers surveyed. In one study conducted at Volta River Authority Hospital in Akosombo, surveyed health providers reported to be satisfied with jobs, team dynamics, and surgical safety [59]. However, they felt that patient communication at discharge (56%) and about medication (31%) could be improved [60]. Another study found that most Ghanaian women with a history of caesarean section find the experience unpleasant (66%) and wish they had been given more information on the risks and benefits of the procedure [61][62]. Addressing job satisfaction is important for ensuring surgical workforce retention, and national policies should design strategies to improve job satisfaction. Poor job satisfaction can lead to workforce emigration as was observed in one study of anaesthesia providers [37].

## Infrastructure, equipment, and supplies

Six studies were identified that described the surgical infrastructure, equipment, and supplies situation in Ghana. One of these studies found that the majority of Ghanaian health facilities have access to reliable water (79%) and electricity sources using the national power grid and backup electricity generators (82%) [62,63]. Studies in other African countries have observed that these basic amenities are often not available [64]. Most district-level facilities do not have consistent access to imaging and operative room infrastructure; however, tertiary facilities have consistent access to these modalities [65]. One area of concern for tertiary facilities in Ghana is the lack of consistent access to oxygen. It is not uncommon for these facilities to run out of their central and backup oxygen supplies, and most facilities do not have an oxygen concentrator on-site [37]. The ongoing COVID-19 pandemic is expected to exacerbate this problem.

The most common causes of equipment and supply deficiency are item absence (52%) and stock-outs (34%). Most stock-outs are untimely due to delayed reimbursements, but medical electronics’ unavailability is often due to breakage [66]. The infrastructure deficit is particularly evident in paediatric surgical services. This disparity is true at all facility levels, especially at regional facilities where hospitals report inconsistent access to emergency supplies, including airway equipment, blood pressure cuffs, cervical collars, and nasogastric tubes [67].

Some studies noted that the unavailability of certain equipment was due to breakage. For example, Ankomah et al., in their nationwide survey of district, regional, and tertiary hospitals, found that 45% of X-rays machines, 27% of mechanical ventilators, 21% of pulse oximeters, and 18% of electronic cardiac monitoring were not available due to breakage [68]. Hsai et al. observed in their research that while a majority of hospitals had established centres to repair large infrastructure, fewer had staff who could fully use the equipment at the hospital [69]. The National Ambulance Service has 199 ambulances and 128 stations [36]. Overall, these studies highlight the need for strengthening surgical infrastructure and the medical supply chain at all levels of care, but especially at the district and regional hospitals.

## Information management

Information management systems of surgical systems are vital for quality improvement and evidence-based decision-making. Our review revealed a scarcity of studies related to information management around SOTA care in Ghana. Five studies related to surgical information management were identified. The Lancet Commission on Global Surgery recommends the tracking of perioperative mortality rates (POMR) at the national level [1]. Our review indicates that this is not currently being tracked in Ghana. There are currently no centralized SOTA databases, so the primary source of information is admission records, patient files, and operative logbooks [37]. These information sources are often incomplete or poorly coded and ineligible [70]. Hospitals with computerized information management systems often have a limited number of staff trained in data entry and retrieval, so data collection and curation depend on their availability [25]. At KATH, a study found that outcomes reporting was often limited to surgical logbooks and departmental end-of-year reports [37]. Deaths were often only recorded when they occurred in the operating room or PACU, and 24-hour or 30-day POMR was not specified [37]. However, the situation is expected to improve with recent implementation of District Health Information and Management Systems at district and regional hospitals. The integration of SOTA key performance indicators such as POMR into these national databases is needed to gather data for policy making and quality improvement.

## Finance

Fifteen studies focused on financing SOTA care in Ghana. Several studies have attempted to estimate the cost of SOTA care as well as the impact of this cost on patients receiving SOTA care. Okoroh et al. estimate the average total direct cost of SOTA care per patient to be US $626, procedures cost US $222 on average, and the average cost of ancillary care/services is US $240 [71]. The average non-SOTA expenditure is US $52, including transportation and lost wages [71].

Health insurance coverage across SSA is generally poor and is as low as 3% and 6% in Nigeria and Mali, respectively [72,73]. Ghana and Rwanda have some of the highest coverage in SSA [74]. In Ghana, most of the direct cost to patients is paid for with out-of-pocket funds (up to 91%), by charitable organizations (up to 60%), and health insurance (up to 14%) [75]. The Ghanaian government launched the National Health Insurance Scheme (NHIS) in 2003 with initial contributions at USD 6–7 per year [76]. In 2013, 68% of the Ghanaian population was NHIS-insured, and although the NHIS reduces catastrophic health expenditure by up to 70%, it does not eliminate out-of-pocket payments even for essential surgical care [71,76,77]. Patients often pay for consultation, laboratory, and pharmacy fees out-of-pocket [76]. The out-of-pocket expenditure of uninsured patients ranges from 1.4–10 times the out-of-pocket expenditures of insured patients [76]. As a result, more uninsured patients experience catastrophic health expenditures than insured patients (29% vs. 7%), but this proportion is steadily decreasing [76].

Health insurance status is directly correlated with quality of care and health outcomes in Ghana. For example, having health insurance increases the likelihood of having a skilled birth attendant during delivery and reduces the likelihood of peripartum maternal and neonatal mortality and morbidity [67]. Despite being NHIS-insured, women are more than twice (aOR: 2.41; 95%CI: 1.77–3.28) more likely to experience catastrophic health expenditure than men [77].

There are social and geographic differences in total health expenditures, out-of-pocket expenditures, and insurance coverage. Wealthier and more educated Ghanaians are more likely to have insurance coverage [67]. Also, the northwest has the lowest health expenditures, while the southwest has the highest health expenditures (Moran Index 0.55, p < 0.01) [50]. While the NHIS has contributed significantly to reducing catastrophic expenditure for healthcare in Ghana, evidence from this review suggests that an update to current policies is needed to ensure further protection for vulnerable people from catastrophic expenditure for SOTA care in Ghana.

## Future directions

This narrative review provides a comprehensive overview of the surgical system in Ghana. To our knowledge, a comprehensive review of the Ghanaian surgical system such as this has not been conducted before. Of value, this narrative review provides information that can be used by the Ministry of Health and the Ghana Health Service and its partners for systems planning programs and formulating policies to strengthen the national surgical system in Ghana. Such policies are needed to prevent the avoidable deaths and morbidity resulting from the high burden of surgical conditions in Ghana as highlighted by this review.

Evidence suggests that despite the high burden of disease from conditions amenable to SOTA care in sub-Saharan Africa (SSA), SOTA care has not been a health priority for many SSA governments. It is estimated that surgical conditions account for more deaths than malaria, HIV/AIDS, and tuberculosis combined. In their analysis of national health strategic plans of SSA countries, Citron et al. found that 19% of these national health priority plans had no mention of surgery or surgical conditions and 65% had five or less mentions of surgery [63]. This implies a limited prioritization of SOTA care in national health plans in SSA.

In order to address the low prioritization of SOTA care in national health plans, nations have been encouraged to develop National Surgical, Obstetric and Anaesthesia Plans (NSOAPs), which comprehensively assesses the surgical ecosystem in the country and details policy strategies to address surgical systems gaps in alignment with the national health plans. To date, seven African countries have developed NSOAPs and several, such as those in the Southern African Development Community (SADC), are in the process of developing similar policies [16,78]. To our knowledge, the Ministry of Health of Ghana is yet to complete a similar policy in alignment with its national health plan, which aims to systematically strengthen the Ghanaian surgical system. This comprehensive narrative review of the Ghanaian surgical system may be used to inform the formulation and implementation of such a policy in Ghana.

This review provides insights into areas of priorities for strengthening the surgical system in Ghana. First, although district hospitals serve as the primary provider of essential and emergency SOTA care in Ghana, they are often understaffed and under-resourced. In developing an NSOAP, the MoH should prioritize the strengthening of workforce and infrastructure capacity, including diagnostic capacity, at the district hospital level. Second, special attention should be placed on increasing the density of skilled SOTA workforce in Ghana. Training capacity should be increased for specializations such as pediatric surgery, trauma, and anaesthesia. Increasing the training capacity of the Faculty of Anaeshesia with the WACS and GCPS could serve to increase anaesthesia density in Ghana and in the region. Third, access to paediatric surgery should be given special attention in the NSOAP to ensure that surgical providers have access to the appropriate resources needed to care for paediatric patients.

Of note, a significant proportion of the studies identified for this narrative review, especially those focused on the quality of SOTA care delivery, were conducted at two major teaching hospitals: Korle Bu Teaching Hospital in Accra and Komfe Anokye Teaching Hospital in Kumasi. Further research should be conducted particularly to assess the quality of SOTA care provided at district and regional hospitals where a majority of essential and emergency surgical care is provided to most Ghanaians. The reporting of POMR at the national level should be prioritized to generate data on quality of surgical care. Such information is needed for the design of interventions and policies focused on improving SOTA care quality at lower levels of care.

## Limitations

While we sought to gather as much literature on the surgical system in Ghana, it is possible that not all relevant studies were retrieved and included in this review. We only surveyed the PubMed database for studies. Other databases and grey literature were not included in our methods. It is therefore possible that some studies might have been omitted and could affect the comprehensiveness of this review. This narrative review, like most such reviews, is likely not reproducible and by nature and method, subject to study selection bias and narrative bias [80]. Nonetheless, our search strategy, unlike a comprehensive systematic review, sought to gather the most essential studies that will allow for a comprehensive overview of the surgical landscape in Ghana over the period. We believe that the studies retrieved and included in this review allowed us to do just that.

## Conclusion

This narrative review represents a comprehensive situation analysis of the SOTA landscape in Ghana according to the LCoGS NSOAP framework. The findings of this review highlight the strengths and weaknesses of the SOTA care delivery system in Ghana and can be used to inform the development of a structured NSOAP as well as programs to strengthen SOTA care access and delivery in Ghana. The comprehensiveness of this situation analysis highlights the methods used as an efficient and cost-effective approach to gathering information on the burden of SOTA diseases and access to SOTA care in Ghana. This review could be used as situation analysis to inform the development of an NSOAP or other surgical-related policies by policy makers in Ghana. Similarly, non-governmental organization and other entities interested in strengthening surgical services in Ghana could use the information provided here to gain a high-level holistic understanding of the surgical system in Ghana to inform program design.

## Paper context

In Ghana, poor access to quality surgical, obstetric, trauma, and anaesthesia care leads to high mortality and disability rates. While there have been several individual studies assessing the surgical system in Ghana, none have systematically and holistically assessed the entire surgical system. This study provides a holistic look at the entire surgical system in Ghana, and the information can be used to inform national policies and programs to strengthen the surgical system in Ghana.
